# SB225002 Promotes Mitotic Catastrophe in Chemo-Sensitive and -Resistant Ovarian Cancer Cells Independent of p53 Status *In Vitro*

**DOI:** 10.1371/journal.pone.0054572

**Published:** 2013-01-24

**Authors:** Meirong Du, Qing Qiu, Andree Gruslin, John Gordon, Miao He, Chi Chung Chan, Dajin Li, Benjamin K. Tsang

**Affiliations:** 1 Laboratory for Reproductive Immunology, Hospital and Institute of Obstetrics & Gynecology, Institute of Biomedical Sciences, Fudan University Shanghai, Medical College, Shanghai, China; 2 Departments of Cellular & Molecular Medicine and Obstetrics & Gynaecology, University of Ottawa, Ottawa, Canada; 3 Chronic Disease Program, Ottawa Hospital Research Institute, Ottawa, Canada; 4 Department of Veterinary Microbiology, University of Saskatchewan, Saskatoon, Canada; 5 WuXi AppTech Co., Ltd., Shanghai, China; 6 Department of Obstetrics & Gynecology, Hainan Medical College Affiliated Hospital, Haikou, China; 7 World Class University (WCU) Biomodulation Major, Department of Agricultural Biotechnology, College of Agriculture and Life Sciences, Seoul National University, Republic of Korea; Rush University Medical Center, United States of America

## Abstract

Recent evidence indicates that CXCR2 signaling is crucial for cancer progression, and its antagonist SB225002 induces apoptosis in Wilms’ tumor cells. Here, we investigated the effect of SB225002 on cell cycle progression and apoptosis induction *in vitro*, using CDDP-sensitive and -resistant OVCA cell lines with different p53 status (wild type, mutant or null). Adenovirus infection of wild-type p53 or transfection of p53 siRNA was used to over-express or knock-down p53. Cell cycle and apoptosis were determined by flow cytometry or Hoechst staining and observation of nuclear morphology. Our data demonstrated that SB225002 induced apoptosis in both wild-type and p53-deficient ovarian cancer (OVCA) cells through alternative mechanisms. SB225002 promoted mitotic catastrophe, as evidenced by the accumulation of mitotic cells with spindle abnormalities, chromosome mis-segregation, multi-polar cell division, multiple nuclei, aneuploidy/polyploidy and subsequent extensive apoptosis. SB225002-induced mitotic catastrophe appeared to be mediated by down-regulation of checkpoint kinase Chk1 and Cdk1-cyclin B activation. In cells expressing wild-type p53 (OV2008 and C13*), SB225002 increased total and phospho-Ser p53 levels, and p53 knock-down decreased SB225002-induced apoptosis, without affecting premature mitosis. These results suggest that SB225002 induces p53-dependent apoptosis, and provokes mitotic catastrophe in p53-independent manner in p53 wild-type cells. Reconstitution with wild-type P53 in P53-null SKOV3 cell attenuated SB225002-induced mitotic catastrophe, suggesting p53 prevented mitotic catastrophe induced by SB225002 in p53-deficient OVCA cells. Finally, the effect of SB225002 could not be prevented by pretreatment with CXCR2 ligand or its neutralizing antibody. The present studies demonstrate for the first time that SB225002 has dual actions in OVCA cells, inducing classic apoptosis through p53 activation and provoking mitotic catastrophe in both p53 wild-type and deficient cells by Chk1 inhibition and Cdk activation. These findings raise the possibility of SB225002 as a new candidate molecule for OVCA therapy independent of the p53 status.

## Introduction

Ovarian cancer (OVCA) is the most fatal gynecological malignancy that has frustrated both clinicians and researchers for several decades. It is the fifth leading cause of cancer-related death among women, with estimated 21,990 newly diagnosed cases and 15,460 deaths occurring in the United States in 2011 (http://seer.cancer.gov/statfacts/html/ovary.html). Although chemotherapy and cytoreductive surgery are currently standard modalities for OVCA treatment, chemo-resistance remains a major cause of chemotherapeutic failure. Various mechanisms have been implicated for chemoresistance, including altered drug transport, enhanced DNA repair and increased tolerance to DNA damage [1], [2], [3]. In addition, mammalian cells exhibit complex cellular responses to genotoxic stress, including cell cycle checkpoint, DNA repair and apoptosis, and gene activation is a critical initial step during the cellular response to DNA damage. Although adjuvant chemotherapy with paclitaxel and cisplatin achieves clinical response in a high percentage of cases, the systemic toxicity severely limits treatment opportunity [4]. Thus, more effective and safe therapeutic strategies are urgently required to combat this disease.

Apoptosis is characterized morphologically by cytoplasmic shrinkage, chromatin condensation and nuclear fragmentation with chromatinolysis [5], and biochemically by caspase activation and permeabilization of the outer mitochondrial membrane [6], [7], [8]. Mitotic catastrophe is a special case of apoptosis, and occurs during mitosis with a combination of deficient cell-cycle checkpoints and cellular damage [9]. DNA damage activates checkpoints to delay cell cycle progression. P53 regulates G1/S transition (G1-checkpoint), while CHK1 prevents the entry of DNA-damaged cells into M-phase (G2-check point). In the case of chemoresistance, DNA damage induced by chemotherapeutic agents fails to arrest the cancer cells in the G1 phase and to promote apoptosis owing mainly to the deficient p53 signaling. However, DNA damage can activate the CHK1 pathway, induce S- and G2-checkpoints arrest [10], [11] and facilitate DNA repair before entry into mitosis (M phase). It is therefore conceivable that agents capable of inducing conventional apoptosis (through DNA damage and P53 activation), and promoting mitotic catastrophe (via CHK1 inactivation and abrogation of G/M arrest) may offer potentially new therapeutic advantages.

Chemokines, a superfamily of small cytokine-like proteins, are known for their pivotal role as regulators in cellular migration, intercellular communications and tumour progression by offering an appropriate tumor microenvironment [12], [13], [14]. CXCR2, a functional receptor for ELR^+^ chemokines, is predominantly expressed in endothelial cells and is a potent promoter of angiogenesis in solid cancers. Evidence indicates that cellular transformation by oncogenic ras induces high expression of all CXCR2 ligands, and contributes to multiple tumor development. In OVCA, ELR^+^ chemokines, such as IL-8, GRO-1 and ENA-78, are significantly up-regulated [15], [16], [17], [18]. The high level of GRO-1 induces the senescence of fibroblasts and promotes malignant transformation of ovarian epithelial cells and OVCA cell growth [19] SB225002 [N-(2-hydroxy-4-nitrophenyl)-N′- (2-bromophenyl) urea] is believed to be a potent selective non-peptide CXCR2 antagonist by inhibiting the binding of both IL-8 and GRO-1 to CXCR2 [20]. Although SB225002 was first developed for the treatment of inflammatory diseases [20], [21], recent studies suggest that retrovirus infections, Wilms’ tumor and Alzheimer’s disease may be potential therapeutic indications for CXCR2 antagonist [22], [23], [24]. SB225002 has been reported to induce apoptosis in Wilms’ tumor cells, although the underlying mechanism is not clear [23].

In the present study, we examined the effect of SB225002 on cell cycle progression and apoptosis induction *in vitro*, using CDDP-sensitive and -resistant OVCA cell lines with different p53 status (wild type, mutant or null). We found that SB225002, independent of CXCR2, induced classic apoptosis through activation P53 and provoked mitotic catastrophe in both P53 wild-type and deficient cells via checkpoint kinase 1 (Chk1) inhibition and Cdk activation. In addition to its inhibitory action of tumor angiogenesis through CXCR2-mediated cell signaling (Matsuo *et al*., 2009), our study demonstrates that SB225002 could be a potential therapeutic agents for chemoresistant OVCA through its action on cell cycle progression and apoptosis independent of receptor-mediated signaling.

## Materials and Methods

### Reagents

Cis-diaminedichloroplatinum (CDDP) and Hoechst 33258 were purchased from Sigma (St. Louis, MO, USA). SB265610 was purchased from Tocris Bioscience (USA). SB225002 was purchased from Calbiochem (San Diego, CA, USA), small inhibitory RNA (siRNA) to p53 were from Cell Signaling Technology (Beverly, CA). Control siRNA was from Dharmacon inc. (Lafayette, CO, USA). Ribojuice siRNA transfection reagent was from Novagen Inc. (San Diego, CA, USA). Primary antibodies for Western blot were rabbit polyclonal anti-PARP, anti-phospho-chk1 (S345) and phospho-p53 (S15; Cell Signaling Technology), mouse monoclonal anti-chk1, anti-caspase 3, anti-phospho-Histone H3 (Serine 10), and anti-p21^WAF1/CIP1^ (Cell Signaling Technology), anti-p53 (DO-1, Santa Cruz Biotechnology, Santa Cruz, CA), anti-IL-8 and GRO-1 (R&D system, Minneapolis, MN) and anti-GAPDH (ab8245, Abcam, Cambridge, UK). The horseradish peroxidase-conjugated secondary antibodies were from Bio-Rad (Hercules, CA, USA). Primary antibodies for immunocytochemistry were rabbit polyclonal Alexa Fluor® 488-conjugated anti-phospho-Histone H3 (Ser 10; Cell Signaling Technology,) and FITC-conjugated anti-β-tubulin (Clone TUB 2.1, Sigma). The monoclonal anti-human CXCR2 -PE, human recombinant IL8 and GRO1 were from R&D System. Pre-stained polyacrylamide gel electrophoresis standards were from BioRad. Adenoviral wild-type p53 and LacZ cDNA were provided by Dr. Ruth Slack (Neuroscience Research Institute, University of Ottawa). ELR-CXC chemokine antagonist CXCL8 (3–72)K11R/G31P (G31P) was from Dr John R. Gordon (University of Saskatchewan, Saskatoon, Canada).

### Cell Culture

Cisplatin-sensitive OVCA cells (OV2008 and A2780s) and their respective resistant counterparts (C13* and A2780cp, respectively), and p53 null SKOV3 cells were cultured as reported previously by our laboratory [25]–[31]. Cells, plated at a density of 5×10^4^ cells/cm^2^, were infected with adenoviral expression vectors or transfected with siRNA or treated with freshly prepared SB225002 under serum-free conditions. Conditioned medium (10 ml) were concentrated with Amicon Ultra-4 centrifugal filter (Millipore Corporation, Billerica, MA) to final volume of 200 µl for subsequent analysis.

### RNA Extraction, cDNA Synthesis and Real-time RT-PCR

Total RNA was extracted (RNeasy Mini Kit. Qiagen) according to manufacture’s instructions. Complementary DNAs were generated by using oligo (dT) primers with MMuLV reverse transcriptase (Retroscript kit; Ambion). Real-time quantitative PCR was performed with the following primers: GRO-1: F: TGCAGCTGTGTCTCTCTTTCCTCT and R: AAAGCTTGCCTCAATCCTGCATCC; IL-8: F: AGCCTTCCTGATTTCTGCAGCTCT and R: AATTTCTGTGTTGGCGCAGTGTGG; CXCR2: F: AGAAGTTTCGCCATGGACTCCTCA and R: AGTGGAAGTGTGCCCTGAAGAAGA; F: GGGGAGCCAAAAGGGTCATCATCT and R: GAGGGGCCATCCACAGTCTTCT for GAPDH. Amplification reaction was performed by using the QuantiTect SYBR Green PCR kit. The thermal cycling conditions included an initial denaturation step (95°, 15 min) and followed by PCR (95° for 15 min, 50 cycles at 95° for 15 sec, 56° for 20 sec, and 72° for 30 sec). PCR products were subsequently melted at 60° for 30 sec and mRNA abundance of IL-8, GRO-1 and CXCR2 were expressed as a ratio to GAPDH values. Every value was compared with that of OV2008.

### Adenovirus Infection, RNA Interference and SB225002 Treatment

SKOV3 cells were infected with adenoviral wild-type p53 or LacZ control (MOI = 10) [25]. Twenty-four hours after infection, cells were treated with SB225002 (750 nM, 24 h or 48 h). OV2008 and C13* cells were transfected with p53 siRNA (100 nmol/L; 36 h) or scramble sequence (control) [26] and then treated with SB225002.

### Western Analysis

Western blotting was performed as previously described [25], [26]. Membranes were incubated overnight at 4°C in primary antibodies (anti-p53 1:10,000; anti-phospho-p53 (S15) 1∶1,000; anti-chk1 1:1,000; anti- phospho-chk1(S345) 1∶1,000; anti-phospho-chk1(S317) 1∶1,000; anti-caspase 3 1:1,000; anti–PARP, 1∶1,000; anti-phospho-Histone 3 (S10; 1∶1,000), anti-GAPDH, 1∶20,000), followed by incubation (RT, 1 h) with horseradish peroxidase– conjugated anti-rabbit or anti-mouse secondary antibody (1∶5,000). Peroxidase activity was visualized with ECL kit (Amersham Biosciences, Piscataway, NJ). Results were scanned and analyzed using Scion Image software (Scion, Inc., Frederick, MD).

### Propidium Iodide Staining and Flow Cytometry

Floating and adherent cell were collected and fixed in 70% ethanol overnight. They were treated with RNase A and incubated with propidium iodide (50 µg/ml; PI) and were analyzed on a Beckman Coulter FC500. For identification of mitotic cells, cells were incubated (2 h, RT) with anti-phospho-histone H3 (Alexa 488, 1∶10, Cell Signaling) and washed with PBS before PI staining. To detect the expression of CXCR2 expression on OVCAs, the cells were harvested and washed in PBS, then immediately stained by a standard immunofluorescence assay with phycoerythrin (PE)–conjugated anti-human CXCR2 mAb or PE-conjugated anti-human isotype antibody (BD Pharmingen). The percent of CXCR2-positive cells was determined by flow cytometry.

### Immunofluorescence

Cells were fixed and permeabilized, then blocked with 2% BSA followed by incubation (overnight, 4°) with FITC conjugated mouse anti-beta-tubulin (1∶50) or FITC-conjugated isotype IgG (negative control). After washing, Hoechst nuclear stain (33258) was added to the cells, which were mounted with Vectashied (Vector, Burlingame, CA, USA). Florescence images were captured by using a Leitz DMRX microscope and analyzed with Adobe Photoshop 7.01 (Adobe, Ottawa, Canada).

### Chromosome Spread Assay

Chromosome spreads were prepared by using standard protocol [32]; DNA was stained with 0.1% SYTOX green, and images were obtained with a Leitz DMRX microscope.

### Assessment of Apoptosis

Attached and floating cells were resuspended in neutral-buffered formalin (4%) containing Hoechst 33258 dye (3.75 ng/ml, 24 h). The percentage of apoptosis was determined by nuclear morphology. At least 400 cells were counted in each group with the counter “blinded” to sample identity to avoid experimental bias [25], [26].

### Statistical Analysis

All results are presented as mean ± SEM of at least three independent experiments. Data were analyzed by two-way ANOVA and Bonferroni posttest was used to determine statistical difference between experimental groups (PRISM software version 5.0, GraphPad, San Diego, CA). Statistical significance was inferred at P<0.05.

## Results

### Expression of CXCR2 and its Ligands IL-8 and GRO-1 in CDDP-sensitive and -resistant OVCA Cells

**Figure 1:**
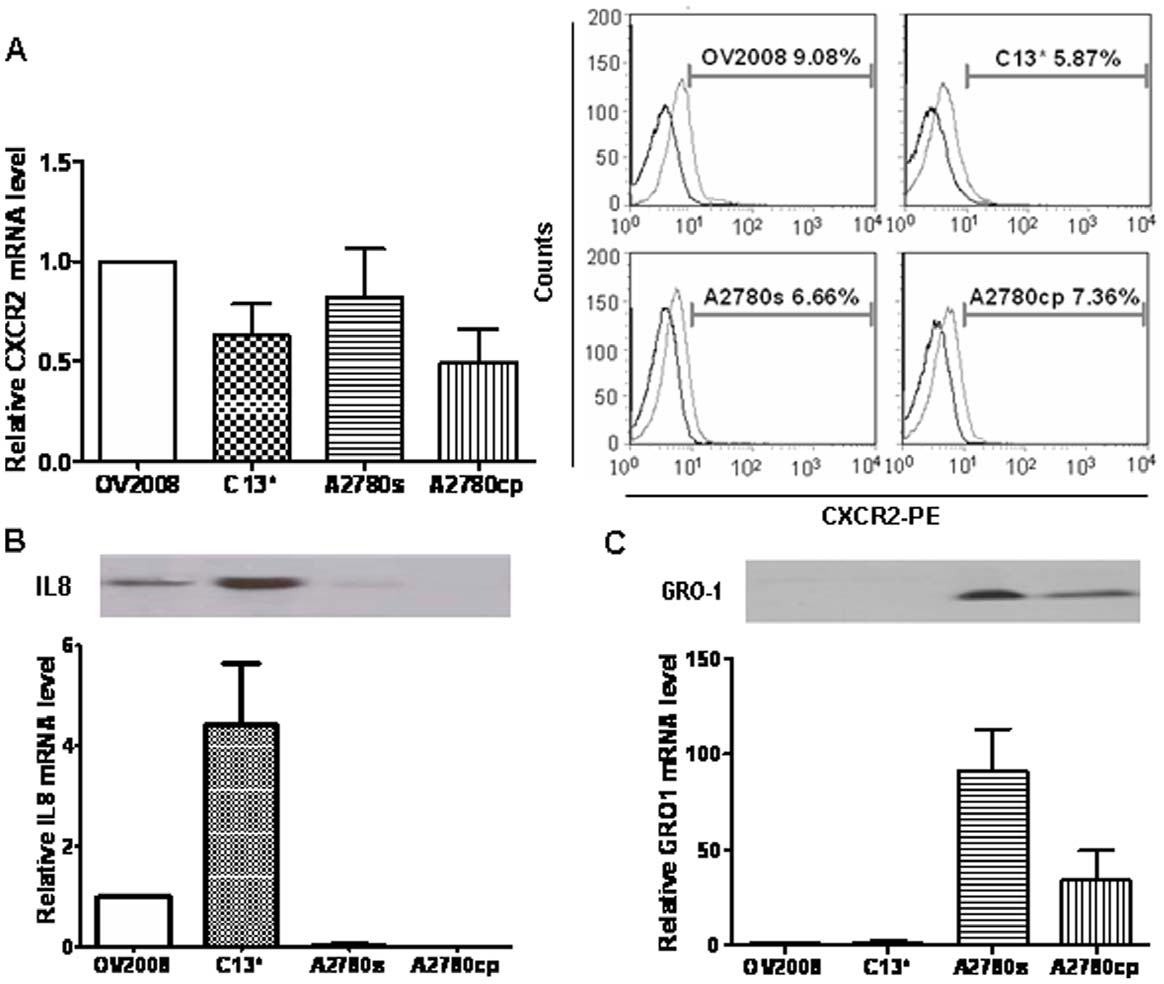
Expression of the chemokine receptor CXCR2 and its ligands IL-8 and GRO-1 in OVCA cells. A: CXCR2 mRNA and protein expression in chemosensitive OVCA cells (OV2008 & A2780s) and their resistant counterpart (C13* & A2780cp, respectively) was determined by qPCR (left) and flow cytometry (right, The darker line represents the isotype control, the lighter line represents the CXCR2-staining cells). B & C: mRNA abundance and protein secretion of IL-8 (B) and GRO-1 (C) [Western blot (upper) and qPCR (lower)]. Data are expressed as means ± SEM of three experiments. Representative pictures of FCM and Western blot are shown.

We first examined mRNA and protein content of CXCR2 and its ligands IL-8 and GRO-1 in two pairs of CDDP-sensitive and -resistant OVCA cell lines. Flow cytometry revealed that CXCR2 was positive in 9.08%, 5.87%, 6.66% and 7.63% of OV2008, C13*, A2780s and A2780cp cells, respectively, and showed no significantly difference between the cell lines. Likewise, there was no significant difference in CXCR2 mRNA abundance (Fig. 1A). In contrast, the IL-8 was mainly expressed in OV2008 and C13* cell lines, while GRO-1 was predominantly in A2780s and A2780cp cells at both mRNA and protein level. IL-8 secretion was 5–fold higher in C13* than in OV2008, whereas GRO-1 secretion was 5-fold higher in A2780s than in A2780cp cells (Fig. 1B, 1C).

### SB225002 Induced Apoptosis and Mitosis in both CDDP-sensitive and -resistant OVCA Cells

**Figure 2:**
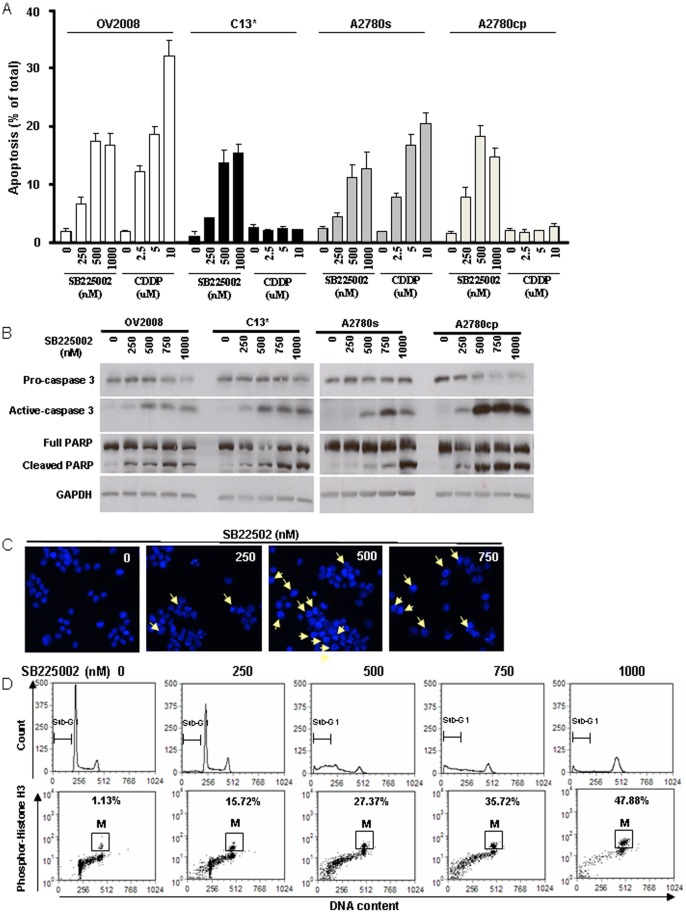
SB225002 induces apoptosis and cell cycle arrest in chemo-sensitive and -resistant OVCA cell lines. A: CDDP (0–10 µM, 24 h) induced apoptosis in chemo-sensitive OV2008 and A2780s but not their resistant counterparts C13* and A2780cp, whereas SB225002 (0–1000 nM, 24 h) induced apoptosis in all cell lines. The OVCAs were treated with different concentration of SB225002 or of CDDP for 24 h. The attached (viable) and floating (apoptotic) cells were pooled, washed and resuspended in neutral-buffered formalin (4%) containing Hoechst 33258 dye (3.75 ng/ml, 24 h). The percentage of apoptosis was determined based on nuclear morphology. At least 400 cells were counted in each group and the counter “blinded” to sample identity to avoid experimental bias. B: SB225002 induced caspase 3 activation and PARP cleavage (Western blot) in all OVCA cells examined. C: SB225002 induced morphological features of apoptosis in OV2008, which was accompanied by mitosis-like cells. D: Flow cytometric analysis of cell cycle distribution of OV2008 after SB225002 treatment at various concentrations (0, 250, 500, 750 and 1000 nM) for 24 h. Cells in M phase were identified with anti-phospho-Histone H3 (P-H3), as shown in the dot plots (lower). The pictures are representative of at least three experiments.

To determine if the CXCR2 antagonist SB225002 would cause cell death in cisplatin-sensitive and -resistant OVCA cells, we assessed apoptosis morphologically in OV2008/C13* (both p53-wild type) and A2780s/A2780cp (p53-wild type and p53 mutant, respectively) cells following SB225002 or CDDP treatment for 24 h. As expected, CDDP induced apoptosis (evident by cytoplasmic shrinkage, chromatin condensation and nuclear fragmentation) in chemo-sensitive cells (OV2008 and A2780s) but not in the resistant cells (C13* and A2780cp). In contrast, SB225002 induced apoptosis in all the cell lines in a concentration-dependent manner (Fig. 2A), an observation supported by caspase 3 activation and PARP cleavage (Fig. 2B). Interestingly, “flower-like” cells with larger and uncondensed chromosomes were evident upon exposure to SB225002 (≥500 nM; Fig. 2C), which is associated with increased cell accumulation at G2/M and sub-G1 phases (Fig. 2D - upper). Most of the increased G2/M phase population was phospho-Histone H3-positive cells (Fig. 2D-low), suggesting that SB225002 increases the number of mitotic cells, possibly by either promoting premature mitosis entry or delaying mitosis exit.

### SB225002 Induced Apoptosis in Wild Type-P53 OVCA Cells through p53 Activation

**Figure 3:**
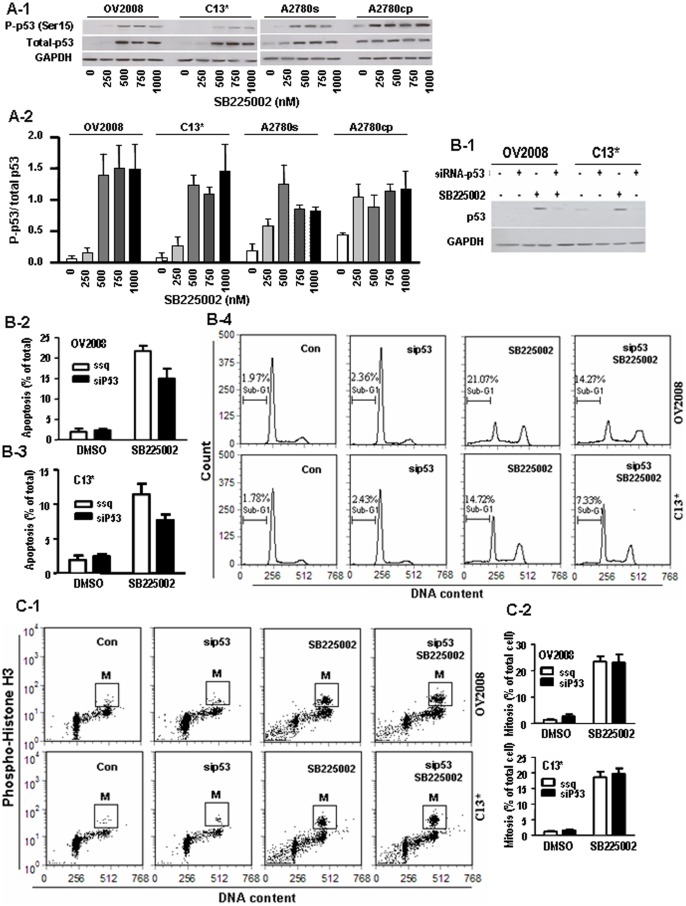
SB225002 induces apoptosis and mitosis in distinct p53-dependent manners. A: SB225002 (0–1000 nM, 24 h) increased total- and phospho-p53 (S15) level in OV2008, C13*, A2780s cells and increased phospho-p53 in A2780cp cells. SB225002 increased the phospho-p53/total-p53 ratio in all cell lines (Western blot). B: Silencing p53 [p53 siRNA (100 nM, 36 h, or scramble siRNA (control)] decreased SB225002 (750 nM, 24 h)-induced apoptosis but not mitosis in OV2008 and C13* cells. C: Silencing p53 had no effect on the cell numbers of entering into mitosis in OV2008 and C13* cells treated with SD225002 at 750 nM. Cell in mitosis were identified by FCM, using anti-phospho-Histone H3 (P-H3).

P53 is essential for the induction of apoptosis in OVCA cells, and is a determinant of CDDP sensitivity [25]. As shown in Fig. 3A, SB225002 (≥500 nM) significantly up-regulated total p53 and phospho-p53 (Ser15) in all the cell lines examined. Silencing p53 by siRNA down-regulated SB225002-induced apoptosis in both OV2008 and C13* cells (Fig. 3B), suggesting that SB225002 induces apoptosis through activating p53. In contrast, the number of phospho-Histone H3-positive cells was not affected by p53 siRNA (Fig. 3C), suggesting the role of p53 in the SB225002-induced apoptosis is not dependent on mitotic cell accumulation.

### SB225005 Induced Accumulation of Pre-mature Mitosis and Subsequent Apoptosis in p53 Wild-type and Deficient OVCA Cells

**Figure 4:**
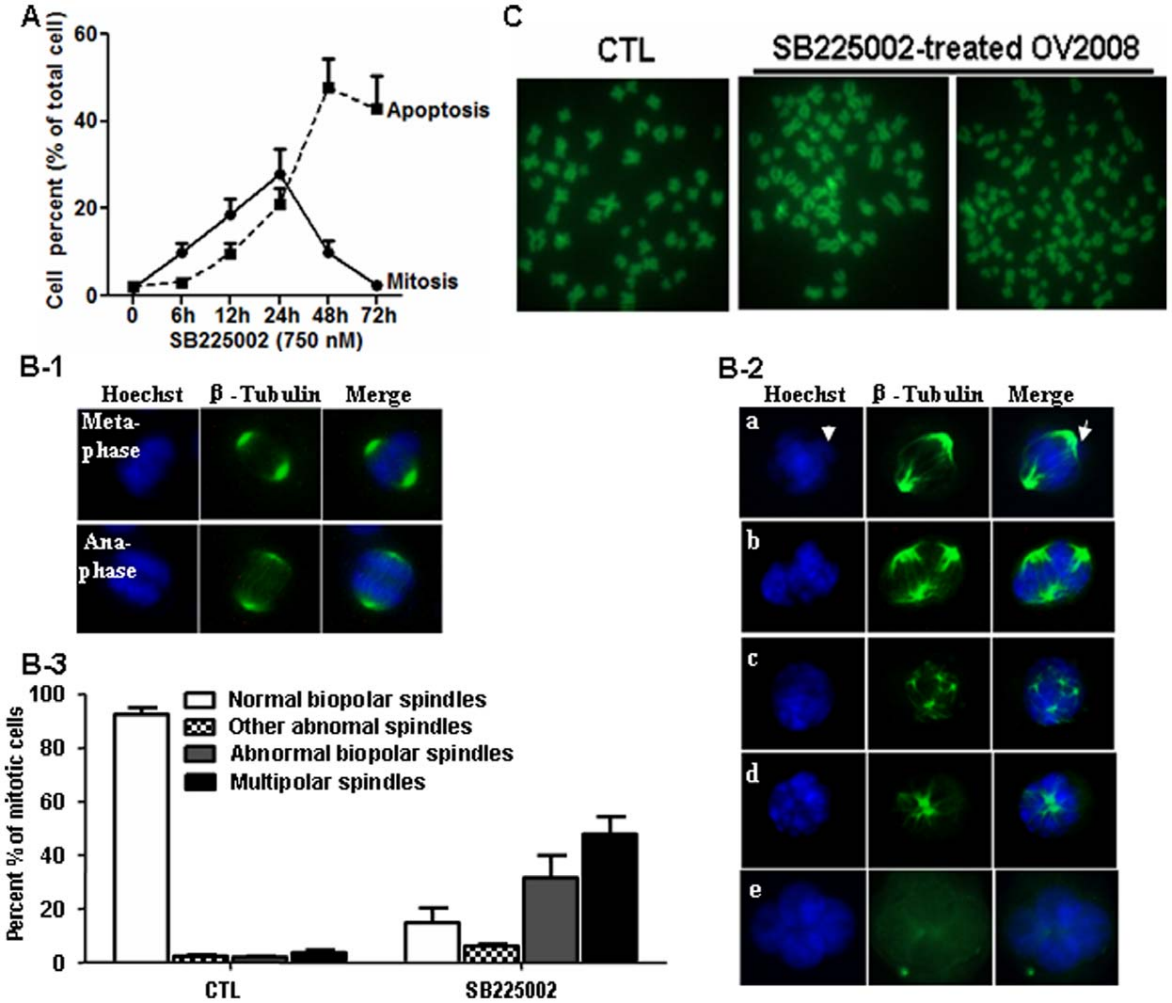
SB225002 induces mitotic catastrophe. A: Time-dependent induction of mitosis (filled circles) and subsequently apoptosis (filled squares) in OV2008 cells by SB225002. B: 24 h after SB225002 treatment, mitotic spindles were visualized with an Alexa Fluor® conjugated to anti-α-tubulin (green) and counterstained with Hoechst (blue). In control cells, normal bipolar spindle was well organized with highly condensed chromosomes aligned along the equatorial plates during meta-phase and sister chromatids segregated at the onset of anaphase (B-1). SB225002-treated cells displayed mitotic aberrations. The frequencies of lagging chromosomal material, multipolar mitotic spindles and multiple nuclei were all increased in SB225002-treated cells (B-2, B-3). C: Chromosome spreads showing SB225002-induced aneuploidy/polyploidy.

Since SB225002 induced mitosis accumulation in both p53-wild-type and deficient OVCA cells, we further determined whether the SB225002-induced mitotic cell accumulation contributed to the apoptosis. SB225002 treatment caused a time-dependent increase in the percentage of mitotic cells (phospho-Histone H3 positive), which began at 6 h, increasing and lasting for 24 h, then declining thereafter. SB225002 induced a late increase in cell population in sub-G1 phase (apoptosis), beginning at 12 h and peaking at 48 h. The time-dependent loss in mitotic cells after 24 h exposure of SB225002 was associated with a marked increase in apoptotic cells, suggesting that SB225002 caused OV2008 cells to arrest in mitosis before progressing onto apoptosis (Fig. 4A).

The morphology of cells undergoing SB225002-induced mitosis was further confirmed for spindle formation, chromatin condensation, and β-tubulin and DNA separation. In control cells, normal bipolar spindle was well organized in an oval shape, highly condensed chromosomes aligned along the equatorial plates during meta-phase and sister chromatids segregated at the onset of anaphase. SB225002 increased the formation of particular types of multipolar spindles, as in pseudobipolar, tripolar and multipolar cells. There was either no chromosome condensation during prophase, or the condensed chromosomes failed to align properly along the equatorial plates during metaphase but instead spread throughout the cells. Cell entering anaphase showed obvious chromosome lagging, indicative of abnormal chromosome segregation in SB-225002-arrested mitotic cells. SB225002 also induced the appearance of multiple nuclei, which was more frequent with longer exposure (Fig. 4B). Aberrant mitosis was associated with aneuploidy/polyploidy cells, as verified by chromosome spreading assay (Fig. 4C). Collectively, our data showed that SB225002 caused abnormal mitotic spindles, altered chromosome segregation in arrested mitotic cells, and resulted in abnormal cell division and formation of multiple nuclei, all of which were indicative of premature mitosis, an event ultimately led to mitotic catastrophe.

### SB225002-induced Mitotic Catastrophe is through Chk1 Down-regulation and Cdk1 Activation

**Figure 5:**
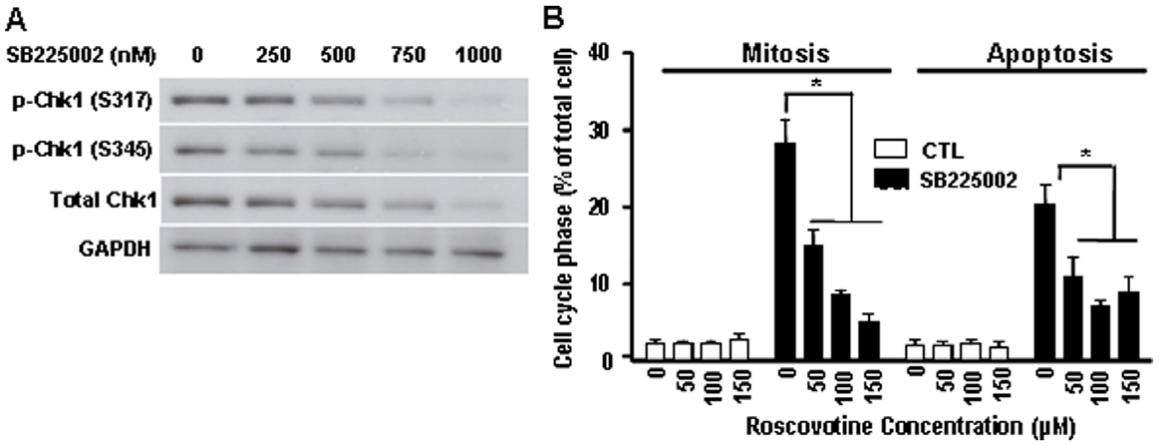
SB225002-induced mitotic catastrophe is associated with Chk1 down-regulation and Cdk1 activation. A: SB225002 (0–1000 nM, 24 h) decreased total and phospho-Chk1 (S317 and S345) levels in OV2008 cells. B: Pretreatment of OV2008 cells with roscovotine (12 h) attenuated SB225002 (750 nM, 24 h)-induced M phase accumulation and reduced subsequent apoptosis. *P<0.05 (compared to SB225002 alone).

DNA damage results in Chk1 activation, which leads to CDC25 degradation, decreased Cdk1 activity and G2 arrest [10]. We have demonstrated that OVCA cells treated with SB225002 exited G2 phase and entered M phase, and underwent premature mitosis, suggesting G2/M phase checkpoint deregulation. Therefore, we investigated whether Chk1 inactivation and Cdk1 activation was involved in the SB225002 action. SB225002 decreased total and phospho-Chk1 (S345) levels in a concentration-dependent manner (Fig. 5A), suggesting that the promotion of premature mitosis entry by SB225002 is associated with Chk1 inhibition and premature Cdk1 activation. This notion was supported by the observation that pretreatment with the Cdk1 inhibitor roscovotine prevented SB225002-induced mitotic cell accumulation and apoptosis (Fig. 5B). Taken together, these results indicate that SB225002 is able to promote apoptosis through mitotic catastrophe most likely through Cdk1 activation.

### Reconstitution of Wild Type P53 in P53-null OVCA Cell Attenuated SB225002-induced Mitotic Catastrophe

**Figure 6:**
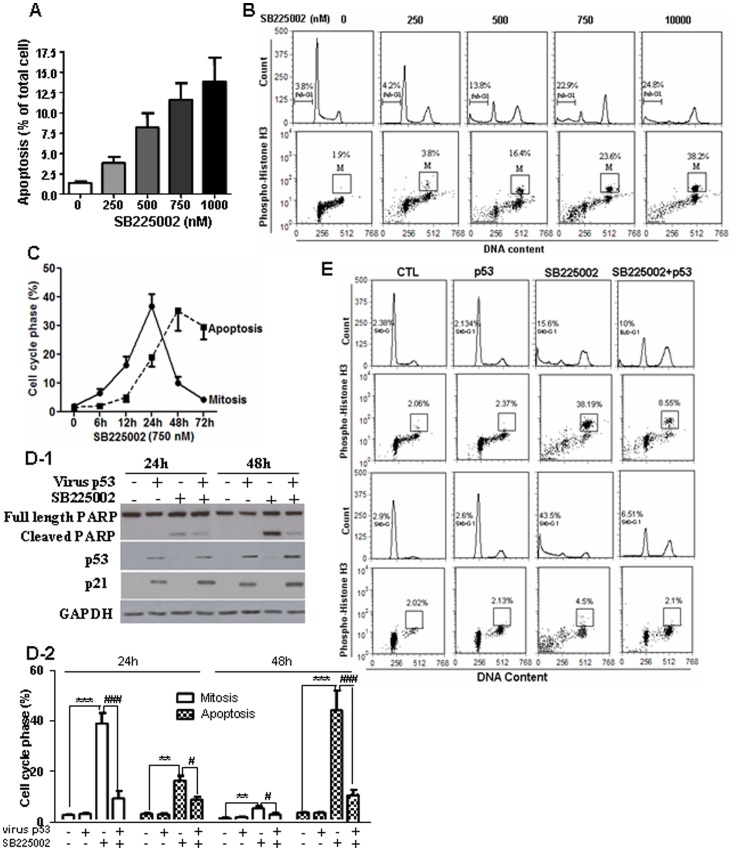
SB225002 induces apoptosis and mitosis in p53-null SKOV3 cells. A: SB225002 (0–1000 nM, 24 h) induced apoptosis in SKOV3 cells (upper). B: SB225002 increased SKOV3 cells in sub-G1 phase (apoptotic cell) and mitosis (phospho-Histone H3-positive; dot plots). C: Time-course of SB225002-induced the mitosis (filled circles) and apoptotic (filled squares) [flow cytometry]. D & E: Adenviral wild-type p53 infection (MOI = 10; adenoviral LacZ to equalize total MOI in each experimental group) of SKOV3 attenuated SB225002 (750 nM; DMSO as control)-induced mitosis and subsequent apoptosis. p53 content and PARP cleavage were assessed at 24 h (Western blot). Cell cycle progression was assessed by flow cytometry. Values are mean ± SEM. *P≤0.05 (compared to DMSO-control). ^#^P≤0.05 (compared to SB225002-treated cells).

To determine if p53 is involved in SB225002-induced mitotic catastrophe, the p53-null ovarian cancer cell line SKOV3 was treated with SB225002. SB225002 induced a concentration-dependent mitotic cell accumulation and apoptosis in SKOV3 cells (Fig. 6A & 6B). The increase in apoptosis was time-dependent and temporally related to SB225002-induced mitosis (Fig. 6 C), suggesting that p53 is unnecessary for SB225002-induced mitotic catastrophe. To further evaluate the action of p53 on SB225002-induced mitotic catastrophe, we examined if reconstitution of wild-type p53 in the p53-deficient cells could change SB225002-induced mitotic accumulation and apoptosis. SKOV3 cells were infected with adenoviral wild-type p53 (confirmed by Western blot; Fig. 6D). As expected, exposure of SB225002 alone induced notably mitosis at 24 h, and marked apoptosis at 48 h. Reconstitution of wild-type p53 markedly prevented SB225002-induced mitosis at 24 h, and attenuated the subsequent apoptosis induced by SB225002 at 48 h (Fig. 6D, 6E). Apoptosis was further confirmed by PARP cleavage at 24 h by Western blot (Fig. 6D). Our results indicate that p53 attenuates SB225002-induced late apoptosis through suppressing the premature accumulation of cells in mitosis. Collectively, these findings suggest that p53 plays opposite role in SB225002-induced apoptosis of OVCA cells, inducing classic apoptosis through p53 activation and preventing apoptosis through mitotic catastrophe.

### SB225002-induced Apoptosis and Catastrophic Mitosis was not through Blocking CXCR2 Receptor Signaling

Since SB225002 is a specific non-peptide antagonist for CXCR2, we then investigated whether the action of SB225002 is mediated through CXCR2. First, we pre-treated OV2008 and C13* cells with different concentrations of IL-8 for 2 h before culturing them with SB225002 (750 nM) for an additional 24 h. Unexpectedly, pretreatment with IL-8 or GRO-1 failed to inhibit SB225002-induced mitosis or apoptosis (Fig. S1A), indicating that SB225002-induced mitosis and apoptosis may not be mediated through CXCR2. In addition, pretreatment of the cells with CXCR2 neutralizing antibody and another antagonist of CXCR1/CXCR2-G31P [33] had no effect on basal or SB225002-induced apoptosis and mitosis in OV2008 and C13* (Fig. S1B and S1C). Moreover, another specific inhibitor of CXCR2, SB265610 had no effect on basal and SB225002-induced apoptosis and mitotic arrest (Fig. S2A & B & C) and p53 activation and Chk1 inhibition (Fig. S2D) in OV2008. Together, these findings support the notion that SB225002 induces apoptosis and mitotic catastrophe in OVCA cells independent of the CXCR2.

## Discussion

In the present study, we have demonstrated that SB225002, a non-peptide CXCR2 antagonist, is effective in inducing apoptosis at checkpoint interphases and mitosis in both CDDP-sensitive and -resistant OVCA cell lines with different p53 status. Treatment of the cancer cells with SB225002 resulted in p53-mediated classical apoptosis but also triggered mitotic catastrophe in p53 wild-type and deficient OVCA cells. SB225002 induced mitotic catastrophe by promoting abnormal mitotic spindles, altering chromosome segregation in arrested mitotic cells, and caused abnormal cell division and formation of multiple nuclei. These changes were abrogated by roscovotine, suggesting that cyclin B/CDK1 had been activated through down-regulation of checkpoint kinase Chk1. The effects of SB225002 were not modulated by pretreatment with CXCR2 ligands or its neutralizing antibody, nor mimicked by other CXCR2 antagonist SB265610 or G31P. Our study provides the first demonstration that SB225002 induced cell death not only through conventional apoptosis but also mitotic catastrophe via a CXCR2-independent manner.

Our study showed that both p53 wild-type and deficient OVCA cells are responsive to SB225002 in the induction of apoptosis, albeit through different mechanisms. P53 can either trigger apoptosis via the activation of intrinsic (mitochondrial) and extrinsic (death-receptor) apoptotic pathway through genomic [33], [34] and non-genomic [27], [28] mechanisms. In addition, p53 can trigger apoptosis via the activation of apoptotic pathway or cell cycle arrest to facilitate DNA repair in DNA damaged cells (an anti-apoptotic process by preventing pre-mature mitosis). Dependent on specific cell types or various environmental stresses, p53 is orientated to exercise specific functions. In p53-wide-type OVCA cells, p53 predominantly activates apoptotic pathways with minimal effect on premature mitosis. This notion is supported by our findings that p53 knockdown reduced apoptosis, but had no apparent effect on the entry into mitosis in cells treated with SB225002 (Figure 3). On the other hand, p53 restoration in p53-null OVCA cells treated with SD225002 resulted in reducing p-histone H3 positive cells and apoptotic cells, suggesting p53 is involved G2/M cycle arrest and prevents catastrophic mitosis (Figure 6).

The significance of our findings on SB225002 relates to its chemotherapy potential with both p53 wide-type and deficient OVCA cells, albeit via different cellular mechanisms. This is in fact one of the major points in this manuscript. Specifically, in p53-wide-type OVCA cells, SB225002 exerts in p53-mediated pro-apoptotic action but had no effect on premature mitosis, but promotes catastrophic mitosis in p53-deficient OVCA cells. Moreover, the present study also demonstrates that SB225002 down-regulates Chk1, a key regulator in G2/M transition, resulting in pre-mature entry of the cells into M phase and subsequent apoptosis.

Evidence that cancer cells with different p53 status (wild-type verse deficient) respond differently to DNA damaging agent and Chk1 inhibitor is conflicting. A large number of studies have shown that combined treatment with a DNA damaging agent and a Chk1 inhibitor preferentially kills P53-deficient cells [35]. Others have demonstrated this combination was effective in p53-wild type cells [36], [37], [38], [39]. In the present study, we have observed that introduction of wild type-p53 to p53-null OVCA cells attenuated SB225002-induced mitotic catastrophe, thus consistent with the notion that restoration of wild-type p53 arrests these otherwise p53 deficient cells at G2 checking point and decreases the number of cells entering M phase.

Mitotic catastrophe is an event followed by DNA damage. Indeed, we observed spindle abnormalities, chromosome mis-segregation, multi-polar cell division, multiple nuclei, and subsequent extensive apoptosis upon SB225002 treatment. While both Chk1 inhibitors (such as UCN-01) and a DNA damaging agent are required for the induction of mitotic catastrophe [40], SB225002 alone is sufficient to promote pre-mature mitosis and subsequent apoptosis. This latter observation suggests a dual role of SB225002 in OVCA cells: genotoxicity and Chk1 inactivation. However, precisely how SB225002 induces DNA damage is currently unclear. Chk1 is involved in the regulation of homologous recombination during DNA repair [41]. In the absence of Chk1, replication forks collapse and replication cannot be resumed correctly, leading to spontaneous DNA damage [36], [42]. Inactivation of Chk1 by SB225002 may in part account for its DNA damaging effect. Indeed, this notion is confirmed by the evidence that Chk1 knockdown by siRNA resulted in DNA damage [43]. It has been reported that UCN-01 enhances CDDP cytotoxicity and apoptosis in OVCA cells [40]. Combination of SB225002 with the genotoxic agent cisplatin failed to enhance SB225002-induced apoptosis in this study (data not shown). These data are consistent with the observation [43] that inhibition of Chk1 does not facilitate the action of DNA damaging agents in reducing cell survival and support the notion that SB225002 alone is able to induce mitotic catastrophe.

The mitotic catastrophe in OVCA cells following SB225002 treatment appeared independent of CXCR2 since pretreatment of OVCA cells with the ligands IL-8 and GRO-1 or the neutralizing antibody of CXCR2 had no effects on SB225002-induced apoptosis, and low CXCR2 was expressed on OVCA cells, and the percentage of apoptosis and mitotic arrest induced by SB225002 was much higher than the expression of CXCR2 on OVCA cells. Furthermore, other specific antagonist of CXCR2 or CXCR1/CXCR2 SB265610 [44] and G31P [45] failed to mimic the action of SB225002, including p53 activation and Chk1 inhibition. While the IC50 of SB225002 in inhibiting IL-8- and GRO-1-induced CXCR2-mediated chemotaxis function is 20 and 60 nM respectively, the concentration of SB225002 needed to induce mitotic catastrophe in this study is at range of 250–1000 nM, implying SB225002 may target other site(s) at higher concentration. CXCR2 knockdown with shRNA resulted in cell cycle arrest at G1/S and G2/M [46], which is in contrast to the abrogation of G2/M arrest by SB225002.

In conclusion, we have demonstrated that SB225002 induces mitotic catastrophe in both CDDP-sensitive and -resistant OVCA cells, and its action is independent of CXCR2 and the p53 status, which could be interpreted as off-target effect with unknown mechanism. In light of the angiogenic and tumorigenic function of CXCR2 in OVCA cells [16], [46], defining the intracellular mechanism of action of SB225002 alone and/or in combination with Chk1 inactivation will shed light on viable approaches in the management of OVCA irrespective of the p53 status of the subject. The challenge for future investigations will be to determine its intracellular target (s) and to establish its in vivo efficacy.

## Supporting Information

Figure S1 **SB225002-induced apoptosis and mitotic catastrophe is independent of CXCR2.** A: Pretreatment of OV2008 cells with IL-8 or GRO-1 failed to influence SB225002-induced apoptosis and mitosis. OV2008 cells were cultured with SB225002 for 24 h after a 2 h -pretreatment with IL-8 or GRO-1 or control (0.5% BSA in PBS). B: Pretreatment of OV2008 cells with a neutralization antibody of CXCR2 (2 h. IgG as control) had no effect on SB225002-induced apoptosis and mitosis in 24 h. C: Pretreatment with G31P (2 h; PBS as control), an inhibitor of CXCR1/CXCR2, failed to inhibit SB225002-induced apoptosis and mitosis in OV2008 cells (24 h; flow cytometric analysis). Data represent mean ± SEM of three experiments.
(TIF)

Figure S2 **SB265610 fails to induce apoptosis and mitosis in OVCA cells.** A: OVCA cells were treated with SB265610 (0–1000 nM) and SB225002 (750 nM) for 24 h and apoptotsis was assessed as above. Unlike SB225002, SB265610 failed to induce apoptosis in all cell lines examined. B: Cell cycle distribution (upper panel) and cell count at M phase (identified with anti-phospho-Histone H3, lower panel) in OV2008 treated with SB265610 (0–1000 nM, 24 h) were determined (flow cytometry). SB265610 had no effect on cell cycle and mitosis. C: Cell number at M phase were counted in OV2008 cells treated with SB265610 (1000 nM) or/and SB225002 (1000 nM) for 24 h. Unlike SB225002, SB265610 failed to increase mitotic cell number in OV2008. D: OV2008 were treated with DMSO, SB265610 (1000 nM), SB225002 (1000 nM) or both for 24 h. The protein level content of PARP (intact and cleaved), p53 [total, phospho-p53 (S15)], Chk1 [total, phospho-Chk1 (S317, S345)] and GAPDH were assessed (Western blot). SB265610 had no influence on the content of these proteins. The pictures are representative of at least three experiments. Data represent mean ± SEM of three experiments.
(TIF)
