# Supplementary figures and images for: SB225002 Promotes Mitotic Catastrophe in Chemo-Sensitive and -Resistant Ovarian Cancer Cells Independent of p53 Status In Vitro
Source: PLoS One. 2013 Jan 24;8(1):e54572. doi: 10.1371/journal.pone.0054572 (PMC3554720; doi:10.1371/journal.pone.0054572)

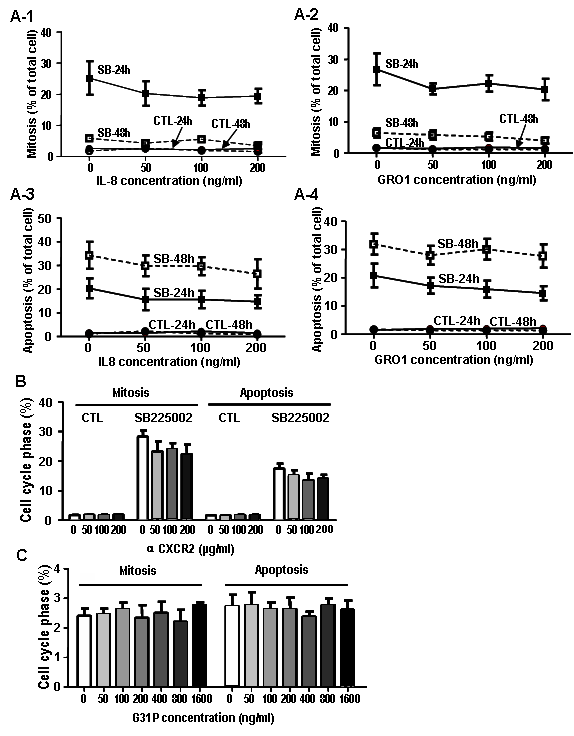

Supplement: Figure S1 — SB225002-induced apoptosis and mitotic catastrophe is independent of CXCR2. A: Pretreatment of OV2008 cells with IL-8 or GRO-1 failed to influence SB225002-induced apoptosis and mitosis. OV2008 cells were cultured with SB225002 for 24 h after a 2 h -pretreatment with IL-8 or GRO-1 or control (0.5% BSA in PBS). B: Pretreatment of OV2008 cells with a neutralization antibody of CXCR2 (2 h. IgG as control) had no effect on SB225002-induced apoptosis and mitosis in 24 h. C: Pretreatment with G31P (2 h; PBS as control), an inhibitor of CXCR1/CXCR2, failed to inhibit SB225002-induced apoptosis and mitosis in OV2008 cells (24 h; flow cytometric analysis). Data represent mean ± SEM of three experiments. (TIF) [file pone.0054572.s001.tif]

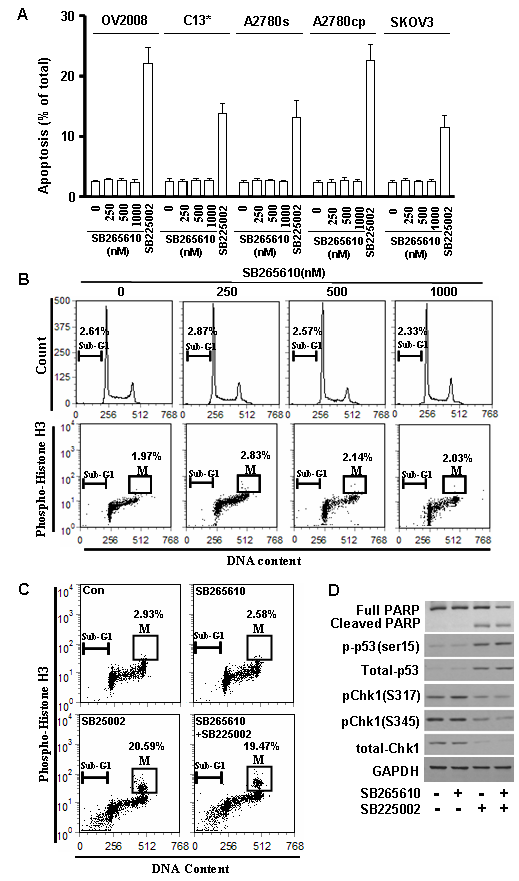

Supplement: Figure S2 — SB265610 fails to induce apoptosis and mitosis in OVCA cells. A: OVCA cells were treated with SB265610 (0–1000 nM) and SB225002 (750 nM) for 24 h and apoptotsis was assessed as above. Unlike SB225002, SB265610 failed to induce apoptosis in all cell lines examined. B: Cell cycle distribution (upper panel) and cell count at M phase (identified with anti-phospho-Histone H3, lower panel) in OV2008 treated with SB265610 (0–1000 nM, 24 h) were determined (flow cytometry). SB265610 had no effect on cell cycle and mitosis. C: Cell number at M phase were counted in OV2008 cells treated with SB265610 (1000 nM) or/and SB225002 (1000 nM) for 24 h. Unlike SB225002, SB265610 failed to increase mitotic cell number in OV2008. D: OV2008 were treated with DMSO, SB265610 (1000 nM), SB225002 (1000 nM) or both for 24 h. The protein level content of PARP (intact and cleaved), p53 [total, phospho-p53 (S15)], Chk1 [total, phospho-Chk1 (S317, S345)] and GAPDH were assessed (Western blot). SB265610 had no influence on the content of these proteins. The pictures are representative of at least three experiments. Data represent mean ± SEM of three experiments. (TIF) [file pone.0054572.s002.tif]
